# The ETS transcription factor GABPA inhibits bladder cancer aggressiveness by repressing extracellular matrix deposition and mechanotransduction signaling

**DOI:** 10.1038/s41419-025-07935-z

**Published:** 2025-08-14

**Authors:** Mingkai Dai, Xiaotian Yuan, Chenxi Sun, Runyuan Han, Magnus Björkholm, Feng Kong, Shengtian Zhao, Dawei Xu

**Affiliations:** 1 Department of Medicine, Division of Hematology, Bioclinicum & Center for Molecular Medicine (CMM), Karolinska Institutet and Karolinska University Hospital Solna, Stockholm, Sweden; 2 Institute of Genome Engineered Animal Models, Shandong Provincial Hospital Affiliated to Shandong First Medical University, Jinan, China; 3 Medical Science and Technology Innovation Center, Shandong First Medical University & Shandong Academy of Medical Sciences, Jinan, China; 4 Department of Hematology, the Second Hospital of Shandong University, Jinan, PR China; 5 Department of Central Laboratory, Shandong Provincial Hospital Affiliated to Shandong First Medical University, Jinan, China; 6 Engineering Laboratory of Urinary Organ and Functional Reconstruction of Shandong Province, Jinan, China; 7 Department of Urology, Qilu Hospital, Cheeloo College of Medicine, Shandong University, Jinan, China

**Keywords:** Cancer microenvironment, Oncogenesis

## Abstract

The ETS transcription factor GABPA exhibits an oncogenic effect by activating telomerase in many cancers; however, some studies imply its tumor suppressive activities. It is thus important to define its different roles in oncogenesis. By examining GABPA-transgenic mice, we unexpectedly observed that Collagen I and III (Col I and III) contents were significantly reduced in murine dermis, which was accompanied by downregulation of prolyl 4-hydroxylase (P4HA2), an enzyme catalyzing collagen folding and fiber stabilization. In bladder cancer (BC) cells, GABPA similarly inhibited Col I and III formation, whereas Col levels increased upon GABPA depletion, revealing its modulation of extracellular matrix (ECM) deposition and stiffness. Mechanistically, GABPA induced miR-30e expression by stimulating *DICER1* transcription, and higher levels of miR-30e consequently targeted P4HA2 for its downregulation. Consistently, P4HA2 overexpression promoted Col formation, cell proliferation, and invasion, while its depletion or the specific P4HA2 inhibitor exerted opposite effects. In tumors derived from GABPA-overexpressed BC cells, atomic force microscope assessment showed that ECM rigidity was significantly reduced, coupled with diminished metastasis in xenografted mice, while P4HA2 overexpression led to stiffer ECM and increased metastasis, counteracting the GABPA effect. Moreover, GABPA knockdown or P4HA2 overexpression promoted YAP1 expression and its nuclear translocation, activating mechanotransduction signaling, through which accelerated proliferation and epithelial-mesenchymal transition occurred. Consistently, high miR-30e and P4HA2 expression were associated with favorable and unfavorable outcomes in BC patients, respectively. Collectively, GABPA inhibits P4HA2 expression and Col formation through the DICER1-miR30e axis, thereby repressing ECM deposition/stiffness and blocking mechanotransduction signaling, which consequently restrains BC aggressiveness. These findings unravel a novel role for GABPA in BC pathogenesis and have biological and therapeutic implications.

## Introduction

The tumor microenvironment (TME) is a complex ecosystem with extracellular matrix (ECM) as one of the key components [1–3]. Cancer cells interact with various stromal cells, which promotes covalent intermolecular cross**-**linkages and massive deposition of ECM proteins such as collagens, thereby creating a rigid ECM [1, 2]. Such dysregulated, rigid ECM contributes to all cancer hallmarks, including proliferation/growth, immortality, death resistance, angiogenesis, invasion, avoidance of immune destruction, and drug resistance [1, 3]. It is, in general, believed that rigid ECM-driven stemness and EMT of cancer cells via biochemical and mechanical signalings play key roles in cancer aggressiveness [2, 3]. Thus, ECM-based cancer therapy has been proposed [1, 4].

Bladder cancer (BC) is the most common urological malignancy worldwide, and more than 90% of them derive from the urothelium [5–7]. Most patients are initially diagnosed as non-muscle-invasive bladder cancer (NMIBC) with longer survival; the newly developed therapeutic strategies and highly reliable approaches for minimal, residual tumor detection and surveillance have further improved patient survival [8–10], however, progression from NMIBC to muscle-invasive bladder cancer (MIBC) still occurs in a fraction of patients, which leads to detrimental outcomes [11]. Molecularly, both NMIBCs and MIBCs can be largely categorized into luminal and basal subtypes based on their unique gene expression signature [7, 12, 13]. The luminal BC subtype is characterized by expression of differentiation-related transcription factors and differentiation biomarkers, while the basal one is poorly differentiated and enriched with stem cell and mesenchymal-like molecules [7, 12–14]. Recent evidence has suggested that the aberrant ECM remodeling is strongly associated with poor patient outcomes in MIBCs [15]; moreover, in both luminal and basal subtypes of BCs, the ECM remodeling alteration leads to BC invasiveness [16, 17]. However, it remains incompletely understood how exactly ECM remodeling is regulated in BC, and conceivably, elucidating the underlying mechanism will contribute to the development of novel ECM-based strategies for BC therapy.

It is well characterized that the hotspot promoter mutations of the *telomerase reverse transcriptase (TERT)* gene (C228T or C250T), occurring in more than 80% of BC tumors, are the key mechanism for telomerase activation and malignant transformation of urothelial cells [18–21], while GABPA, the ETS transcription factor, stimulates the mutated TERT promoter for its transcription [22]. Although GABPA-mediated TERT induction and telomerase activation are required for BC development/progression, we recently unraveled that GABPA itself inhibited BC cell proliferation and invasion, suggesting its tumor suppressive function [10]. To comprehensively dissect the role of GABPA in tumorigenesis, we created a GABPA-transgenic (GABPA-T) mouse model. By examining the in vivo physiological alterations in GABPA-T mice, we unexpectedly observed that GABPA overexpression profoundly inhibited collagen formation. As described above, the aberrant ECM remodeling drives BC aggressiveness, while collagen enrichment is the key determinant for the tumor ECM property, which, together with our recent observations, inspired us to explore whether GABPA exerts its inhibitory effect on BC by regulating ECM remodeling and the mechanotransduction pathway. Our findings reveal that GABPA lowers collagen levels by downregulating the expression of prolyl 4-hydroxylase (P4HA2), an enzyme catalyzing the formation of 4-hydroxyproline residues for proper collagen folding and fiber stabilization [23]. GABPA depletion led to increased collagen synthesis, inducing stiffer ECM accompanied by aggressive BC phenotypes.

## Methods

“Methods section” is detailed in Supplementary materials.

## Results

### Collagen I and III (Col I and III) levels are significantly reduced in GABPA-T mice

**Fig. 1:**
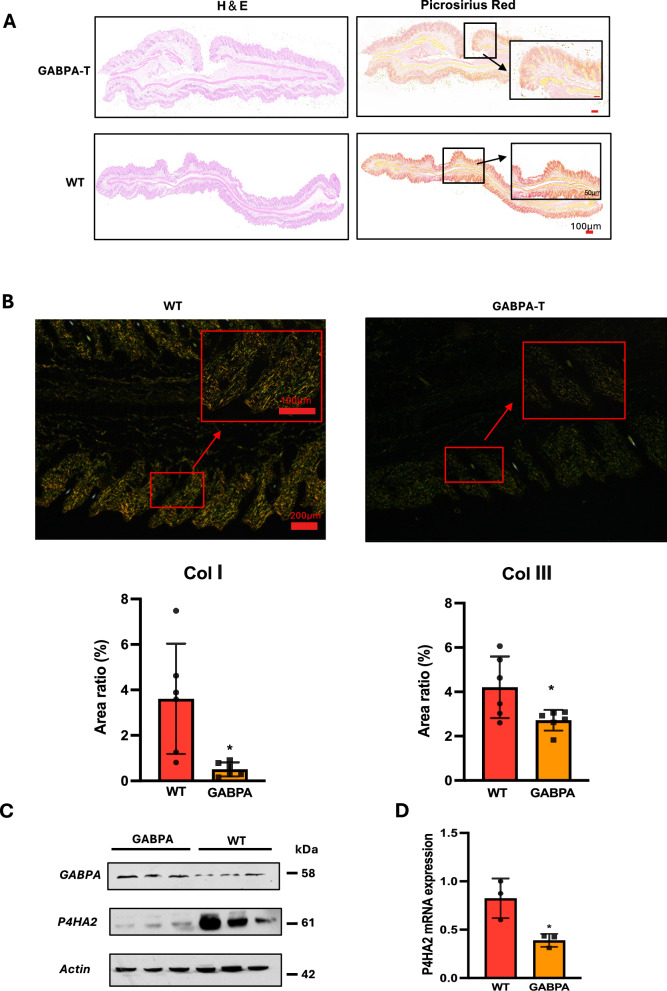
GABPA inhibits collagen formation and P4HA2 expression in mice. **A** Dermis from wild-type (WT) and GABPA-transgenic C57BL/6 mice (GABPA-T) was stained with H & E (left) and Picrosirius Red (PSR) (right), respectively. Representatives are shown. **B** Polarization analysis of PSR staining using a NIKON Eclipse ci upright microscope. Under a polarized microscope, Col I is thick fibers orange-yellow or bright red fibers, whereas Col III is thin, green fibers (Top). Representatives are shown. The quantification of Col I and III in dermis from WT and GABPA-T mice was expressed as area ratio (%) (Bottom). Each group included 3 mice, and two slides from each mouse were analyzed. **C** and **D** P4HA2 downregulation mediated by GABPA. Skin tissues from 3 WT and 3 GABPA-T mice were analyzed for P4HA2 protein (**C**) and mRNA (**D**) levels using immunoblotting and qRT-PCR, respectively. * Indicates *P* < 0.05.

GABPA has long been shown as an oncogenic driver, and especially the recent identification of its role in telomerase activation has led to the proposal to target it for cancer therapy. However, our findings reveal inhibitory effects of GABPA on the progression of several cancer types [10]. To comprehensively investigate the physiological/pathological (oncogenic) roles of GABPA, we generated GABPA-T mice. We first examined organs/tissues from wild-type (WT) and GABPA-T mice to see whether GABPA overexpression leads to physiological alterations. When mouse dermis was compared, we unexpectedly observed that collagen fiber density was significantly lower together with fiber disorganization in GABPA-T mice, as shown by Picrosirius Red (PSR) staining (Fig. 1A). Further polarization analysis confirmed decline in Col I and III levels in GABPA-T mouse dermis and the reduction of Col I levels was more pronounced than that of Col III (Fig. 1B). Given these observations, we probed the underlying mechanism by determining a panel of molecules associated with collagen formation/maturation, and found that the expression of P4HA2, the enzyme catalyzing the formation of 4-hydroxyproline residues, was significantly downregulated in dermis derived from GABPA-T mice (Fig. 1C and 1D). The findings above demonstrate that overexpressed GABPA plays an inhibitory role in Col formation and ECM deposition.

### GABPA inhibits P4HA2 expression and Col formation in BC cells

**Fig. 2:**
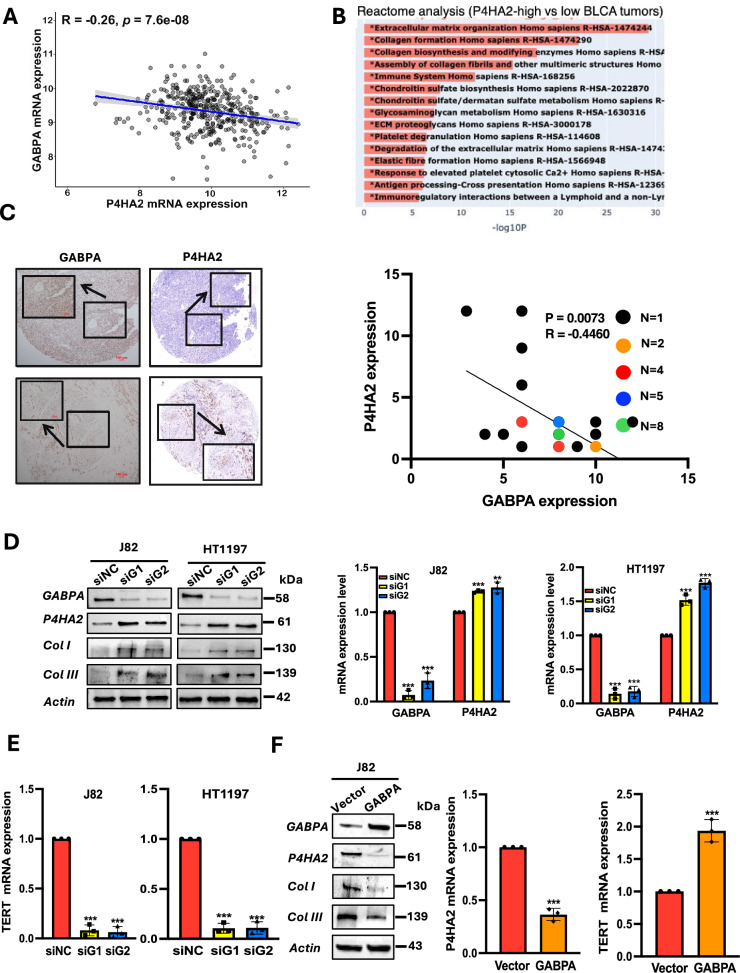
GABPA is anti-correlated with P4HA2 expression in primary BC tumors and downregulates P4HA2 and collagen levels in BC cells. **A** GABPA and P4HA2 mRNA expression is inversely correlated with each other. RNA-seq data from the TCGA BLCA tumors were analyzed. **B** Reactome analysis reveals the enrichment of ECM pathways in P4HA2-high BLCA tumors. The TCGA BLCA tumors were categorized into P4HA2-high and low groups using a median expression value as a cutoff. **C** GABPA and P4HA2 protein levels are inversely correlated with each other. Primary BC tumors were examined for their GABPA (45) and P4HA2 (34) protein levels using immunohistochemistry (IHC). Left: Representative images and right: the inverse correlation between GABPA and P4HA2 protein levels, as assessed by IHC. **D** GABPA depletion results in upregulation of P4HA2, Col I, and Col III in J82 and HT1197 cells. Cells were treated with GABPA siRNAs (siG1 and siG2) and then analyzed for P4HA2, Col I, and Col III levels. **E** TERT expression is downregulated in J82 and HT1197 cells with GABPA knockdown. **F** GABPA overexpression downregulates P4HA2, Col I, and Col III, while stimulating TERT expression in J82 cells. *, **, and *** indicate *P* < 0.05, 0.01, and 0.001, respectively.

Our recent study unravels that GABPA inhibits BC progression, and thus, the findings obtained from GABPA-T mice prompt us to determine whether GABPA inhibits ECM remodeling by regulating P4HA2 expression in BC. Toward this end, we first examined the BC tumors for the correlation between GABPA and P4HA2 mRNA expression in the TCGA BLCA cohort. As shown in Fig. 2A, their mRNA levels were significantly anti-correlated. These BC tumors were then divided into P4HA2-high and low groups using a median mRNA value as the cutoff, and Reactome analysis was then carried out. In P4HA2-high tumors, the top enriched pathways included ECM organization, collagen formation, collagen biosynthesis and modifying enzymes, and assembly of collagen fibrils and other multimeric structures (Fig. 2B). To further determine whether GABPA and P4HA2 are anti-correlated at the protein level, we performed immunohistochemistry (IHC) staining of 45 primary BC tumors. Consistent with the mRNA results obtained from the TCGA BLCA tumors, a strong inverse correlation between them was observed (Fig. 2C). Moreover, 15 of these primary tumors were further analyzed for their GABPA, P4HA2, and Col I levels using multiplex immunofluorescence (IF) staining with Tyramide signal amplification. GABPA was anti-correlated with not only P4HA2 (*R* = −0.67, *P* = 0.006) but also Col I (R = −0.69, *P* = 0.004) (Fig. S1).

To directly determine the effect of GABPA on P4HA2 expression, we manipulated GABPA expression in BC-derived cells and then analyzed P4HA2 expression. BC-derived cells J82 and HT1197 were treated with GABPA-specific siRNAs to inhibit its expression (Fig. 2D). Upon GABPA knockdown, significant upregulation of P4HA2 expression at both mRNA and protein levels occurred in these cells (Fig. 2D). The increased Col I and III levels occurred simultaneously (Fig. 2D). In addition, these cells harbor mutated TERT promoters, and therefore, TERT expression was assessed, too. As expected, GABPA inhibition led to a sharp decline in TERT mRNA levels (Fig. 2E). In contrast, ectopic GABPA expression in these cells inhibited the P4HA2, Col I, and III levels, whereas TERT expression was promoted (Fig. 2F). Taken together, GABPA regulates P4HA2 expression and collagen formation or ECM deposition in BC cells, consistent with the observation in dermis from the GABPA-T mouse.

### P4HA2 and GABPA exert opposing effects on in vitro BC cell invasion and proliferation

**Fig. 3:**
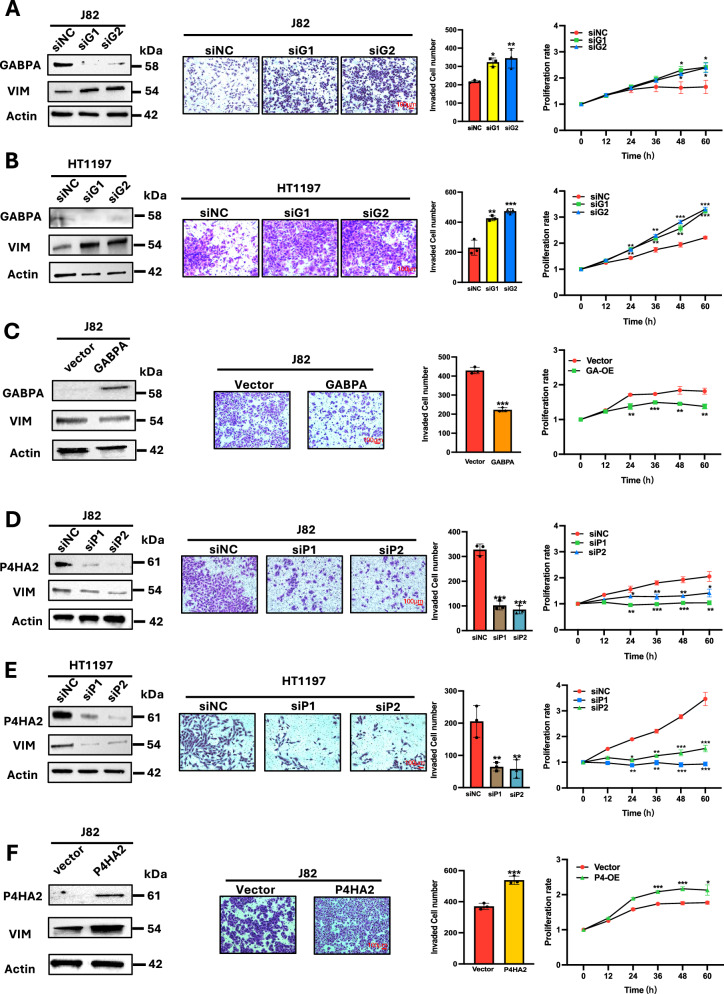
GABPA and P4HA2 exert opposing effects on the invasion and proliferation of BC cells. **A** and **B** GABPA inhibitions promote vimentin (VIM) expression, invasion, and proliferation of BC cells. J82 and HT1197 cells were depleted of GABPA and then analyzed for their vimentin expression (left), invasion (middle), and proliferation. **C** GABPA overexpression (GA-OE) inhibits VIM expression, invasion, and proliferation in J82 cells. J82 cells stably expressing ectopic GABPA were analyzed for their vimentin expression (left), invasion (middle), and proliferation. **D** and **E** P4HA2 knockdown inhibits vimentin (VIM) expression, invasion, and proliferation of BC cells. J82 and HT1197 cells were depleted of P4HA2 using siRNAs and then analyzed for their vimentin expression (left), invasion (middle), and proliferation. **F** P4HA2 overexpression (P4-OE) promotes VIM expression, invasion, and proliferation in J82 cells. J82 cells stably expressing ectopic P4HA2 were analyzed for their vimentin expression (left), invasion (middle), and proliferation. *, **, and *** indicate *P* < 0.05, 0.01, and 0.001, respectively. GA & P4: Cells overexpressing both GABPA and P4HA2.

We then sought to determine the functional effect of P4HA2 and GABPA on BC cells. GABPA depletion significantly promoted invasion and proliferation of J82 and HT1197 cells (Fig. 3A, B), consistent with our previous observations [10]. In contrast, the ectopic introduction of GABPA into J82 cells resulted in inhibition of both invasion and proliferation (Fig. 3C). These GABPA-mediated effects were similarly observed in SW1710 cells (Fig. S2A–G). To determine whether the altered invasion ability of these cells with GABPA manipulation resulted from EMT, we analyzed the expression of vimentin (VIM), a specific mesenchymal marker. Indeed, GABPA depletion and overexpression promoted and inhibited VIM expression in all tested BC cells (Fig. 3A–C, and Figs. S2 and S3), respectively, demonstrating the EMT involvement in GABPA-regulated cell invasion. We next determined the P4HA2 effect using these same cells. P4HA2 knockdown led to diminished cell invasion and proliferation, while its overexpression accelerated cellular division and enhanced invasion (Fig. 3D–F), which was accompanied by down- and upregulated VIM expression, respectively (Fig. 3D–F, and Figs. S2 and S3). In addition, when J82 cells were treated with 1,4-dihydrophenonthrolin-4-one-3-carboxylic acid (1,4-DPCA), a P4HA2 enzyme inhibitor, their invasive ability was significantly reduced in a dose-dependent manner (Fig. S2H).

**Fig. 4:**
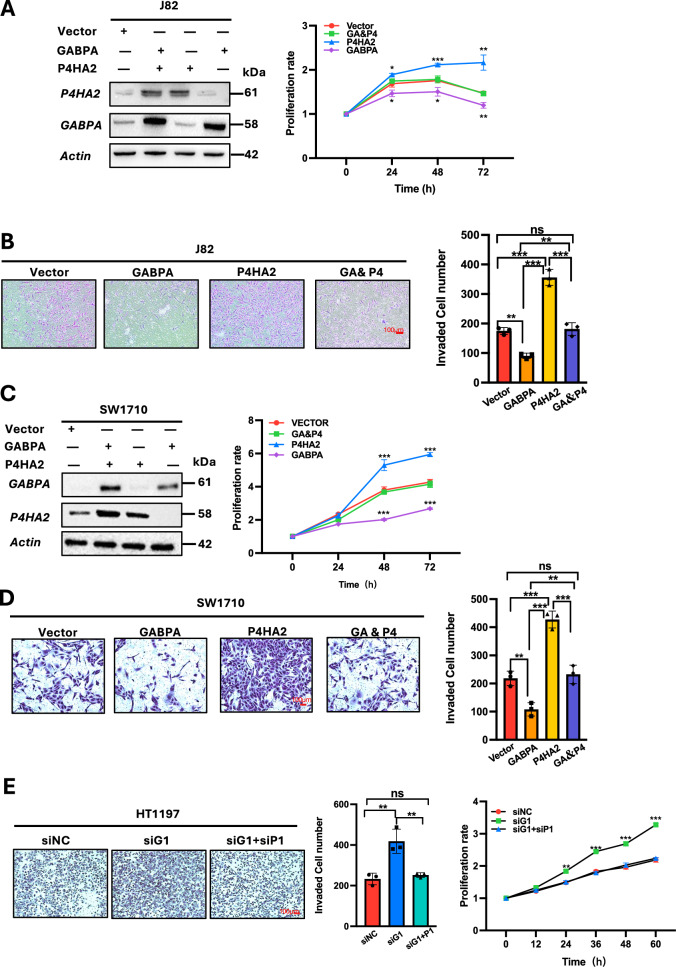
GABPA effects on proliferation and invasion are abolished by P4HA2 in BC cells. **A** and **B** P4HA2 overexpression counteract the proliferation and invasion inhibition resulting from ectopic GABPA expression in J82 cells. J82 cells expressing ectopic GABPA, P4HA2, or both were analyzed for cell proliferation and invasion. **C** and **D** P4HA2 overexpression counteract the proliferation and invasion inhibition resulting from ectopic GABPA expression in SW1710 cells. SW1710 cells expressing ectopic GABPA, P4HA2, or both were analyzed for cell proliferation and invasion. GA & P4: Cells overexpressing both GABPA and P4HA2. **E** Enhanced invasion and proliferation of HT1197 cells by GABPA depletion is abolished by P4HA2 knockdown. HT1197 cells were transfected with GABPA siRNA (siG1) and siG1 plus P4HA2 siRNA (siP1), respectively, and cell proliferation and invasion were then analyzed. *, **, and *** indicate *P* < 0.05, 0.01, and 0.001, respectively.

Given these opposite effects on BC cells, we further sought to probe the functional relationship between GABPA and P4HA2. The GABPA overexpression-mediated inhibitory effect on proliferation and invasion was counteracted by ectopic P4HA2 expression in J82 cells (Fig. 4A). Similar results were obtained from SW1710 cells with GABPA and P4HA2 manipulation (Fig. 4B). In HT1197 cells with high levels of GABPA expression, we transfected these cells with GABPA siRNA and observed significant increases in cell invasion and proliferation. However, simultaneous treatment of cells with both GABPA and P4HA2 siRNAs completely abolished the effect mediated by GABPA inhibition (Fig. 4E). These results collectively suggest that P4HA2 is a downstream effector of GABPA.

### GABPA-mediated inhibition of in vivo BC metastasis is abolished by P4HA2

**Fig. 5:**
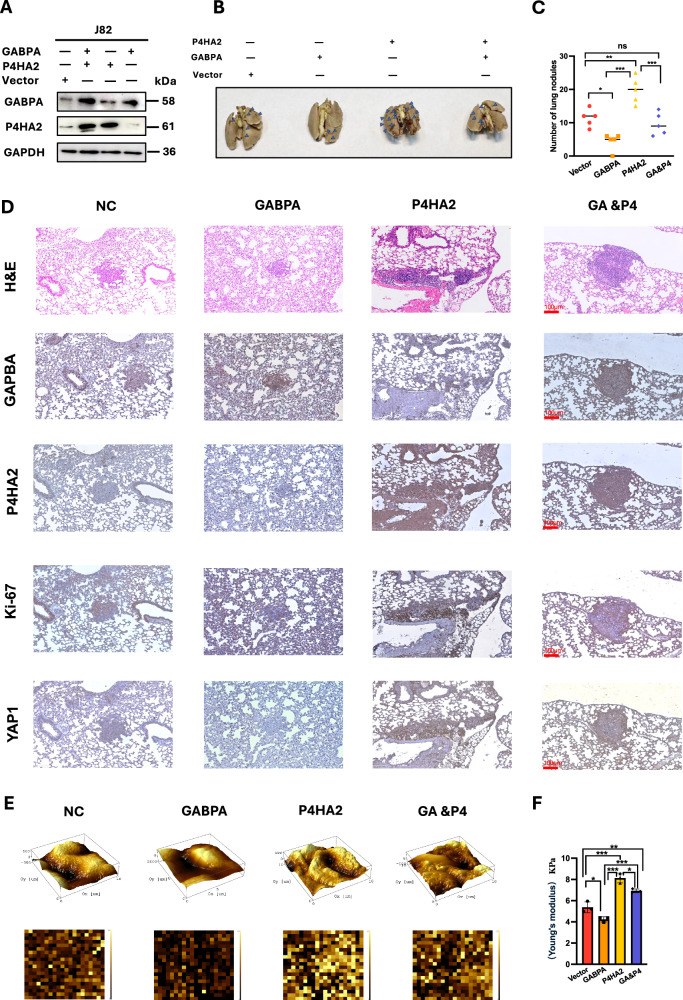
GABPA and P4HA2 effects on in vivo metastasis are associated with tumor ECM stiffness in mouse xenograft models. **A** J82 cells expressing ectopic GABPA (J82/GABPA), P4HA2 (J82/P4HA2), or both (J82/GABPA/P4HA2) and control cells with empty vectors (J82/vector) were verified by immunoblotting. **B** and **C** Metastasized tumor numbers in mouse lungs from each group. Tumor foci were indicated using blue arrows. **D** Tumors from each group were analyzed for GABPA, P4HA2, Ki67, and YAP1 using immunohistochemical staining. **E** and **F** GABPA reduce while P4HA2 increases tumor ECM stiffness in metastasized tumors. **E** Representative images under an atomic force microscope (AFM) in each group. **F** Quantification of ECM stiffness (expressed as Kpa). *, **, and *** indicate *P* < 0.05, 0.01, and 0.001, respectively.

We next determined the in vivo effect of GABPA and P4HA2 on metastasis. For this purpose, J82 cells were modified to stably overexpress GABPA, P4HA2, and both, respectively, and these different cells were then injected into nude mice via the tail vein to set up tumorigenic xenografts (Fig. 5A). Mice were killed 8 weeks post-injection, and their lungs were collected to evaluate the tumor cell seeding capacity. All these injected cells formed tumor foci in lungs (except one mouse injected with J82/GABPA cells), but their numbers differed substantially (Fig. 5B, C). GABPA-overexpressed cells generated the fewest foci, while P4HA2 overexpression induced 2-fold higher numbers of tumor cell seeding, compared to control J82 cells with an empty vector (J82/vector) (Fig. 5B, C). The tumor number in lungs derived from J82 cells overexpressing both GABPA and P4HA2 was almost the same as that of J82/vector cells. IHC analyses demonstrated appropriate expression levels of GABPA and P4HA2 in tumors from corresponding J82 cells (Fig. 5D). In addition, J82/P4HA2-cell-originated tumors exhibited the strongest Ki67 and YAP1 staining, indicating accelerated cell proliferation, whereas GABPA-cell-formed tumors expressed substantially lower levels of Ki67 and YAP1 (Fig. 5D), highly consistent with the in vitro results.

### GABPA reduces while P4HA2 increases the ECM stiffness of metastatic tumors in xenograft models

Based on the cellular experiments, GABPA inhibits P4HA2 expression, thereby resulting in reduced Col I and III formation and maturation, and we thus sought to assess whether these effects contributed to altered tumor ECM stiffness. Toward this end, we carried out atomic force microscopy (AFM) assays of metastatic tumor ECM derived from J82 cells with the manipulation of GABPA and/or P4HA2 expression described above. Compared to tumors derived from control J82-vector cells, GABPA overexpression significantly lowered ECM stiffness, whereas ECM in tumors overexpressing P4HA2 exhibited highly increased stiffness (Fig. 5E, F). In tumors overexpressing both GABPA and P4HA2, the ECM stiffness was significantly weaker than that in J82-P4HA2 tumors but still higher than J82-vector tumors. The stiffness in those tumors is largely consistent with their metastatic capacity, as assessed by foci formation in mouse lungs (Fig. 5B, C).

### GABPA inhibition or P4HA2 overexpression induces YAP1 expression and its nuclear translocation in BC cells

**Fig. 6:**
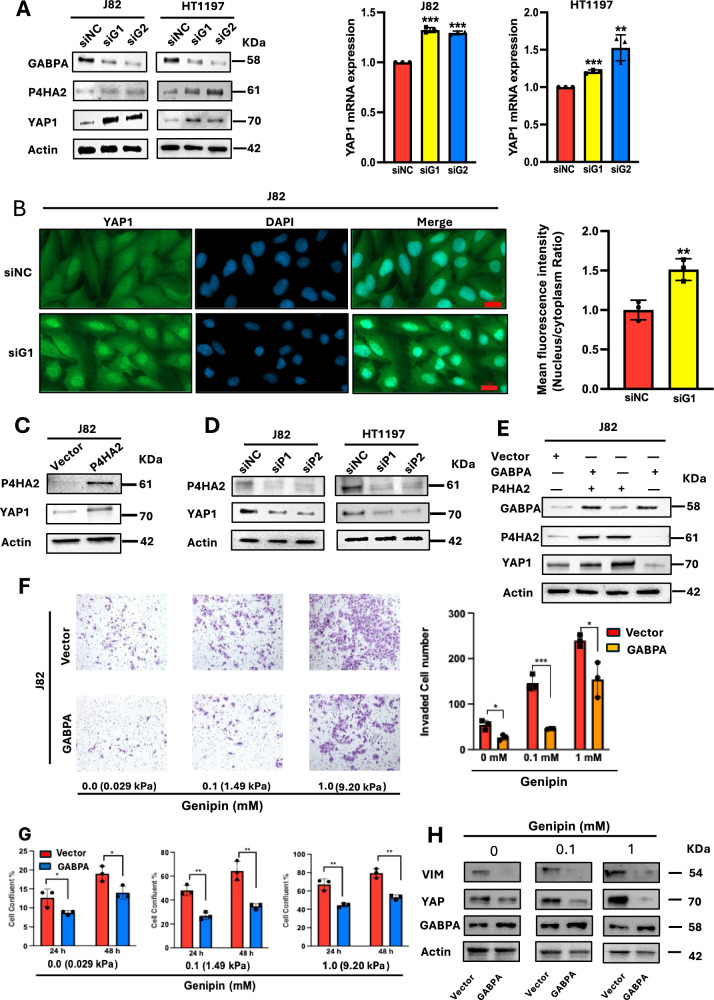
P4HA2 and GABPA regulate YAP1 activation/expression in opposing manners in BC cells, which mimics stiff matrices. **A** YAP1 expression is upregulated in GABPA-depleted BC cells. J82 and HT1197 cells depleted of GABPA were analyzed. **B** GABPA inhibition promotes the nuclear accumulation of YAP1 in J82 cells. YAP1 was stained using its specific antibody conjugated with immunofluorescence. The ratio of immunofluorescence intensity between the nucleus and cytoplasm was calculated to estimate the subcellular distribution of YAP1. **C** P4HA2 overexpression stimulates YAP1 expression in J82 cells. **D** P4HA2 knockdown inhibits YAP1 expression in J82 and HT1197 cells. Cells were transfected with two P4HA2 siRNAs (siP1 and siP2) and then analyzed for YAP1 protein levels using immunoblotting. **E** P4HA2 overexpression abolishes YAP1 downregulation mediated by ectopic GABPA expression in BC cells. J82 cells expressing ectopic GABPA, P4HA2, and both were analyzed for YAP1 protein levels using immunoblotting. **F**, **G** Stiff matrices promote invasion and proliferation of J82 cells, which are attenuated by GABPA overexpression. Control and GABPA-overexpressed J82 cells were cultured on genipin-containing gels with different concentrations (0, 0.1, and 1.0 mM), and then analyzed for their invasion and proliferation. **H** Stiff matrices upregulate YAP1 and VIM expression in J82 cells. *, **, and *** indicate *P* < 0.05, 0.01, and 0.001, respectively.

It is well established that tissue or tumor stiffness-mediated mechanical signals are transduced by mechanosensitive machinery, including a key transcriptional coactivator, YAP1, in the Hippo pathway [24–26]. Thus, we sought to examine whether GABPA and P4HA2 manipulation lead to altered YAP1 expression or subcellular distribution. In both J82 and HT1197 cells, YAP1 expression at mRNA and protein levels was significantly upregulated upon GABPA knockdown, which was accompanied by enhanced P4HA2 expression (Fig. 6A). In J82 cells depleted of GABPA, we further performed immunofluorescent staining of YAP1, and observed its significant increase in nuclear translocation (Highly increased ratio between nucleus and cytoplasm) (Fig. 6B). On the other hand, P4HA2 overexpression increased the level of YAP1 while its knockdown inhibited YAP1 expression (Fig. 6C, D). Moreover, GABPA-mediated YAP1 downregulation was abolished by ectopic P4HA2 expression (Fig. 6E).

### Stiff matrices mimic the P4HA2-mediated phenotypic changes but are attenuated by GABPA

Genipin-mixed Col gels, recently established by Ishihara et al. [27], were used to culture BC cells. Genipin concentrations at 0.0, 0.1, and 1.0 mM generated Young’s moduli of gels with 0.029, 1.49, and 9.20 kPa, respectively. Control J82 cells grown on 1.0 mM-containing genipin gels (9.2 kPa) exhibited significantly enhanced invasion and proliferation compared with those on gels with 0 and 0.1 mM of genipin (Fig. 6F, G), which was coupled with upregulation of VIM and YAP1 expression (Fig. 6H). These results are in accordance with P4HA2-mediated changes in both phenotypes and gene expression. On the other hand, GABPA overexpression attenuated all the changes resulting from stiff matrices in J82 cells (Fig. 6F–H).

### GABPA-mediated miR-30e expression targets P4HA2 for its downregulation in BC cells

**Fig. 7:**
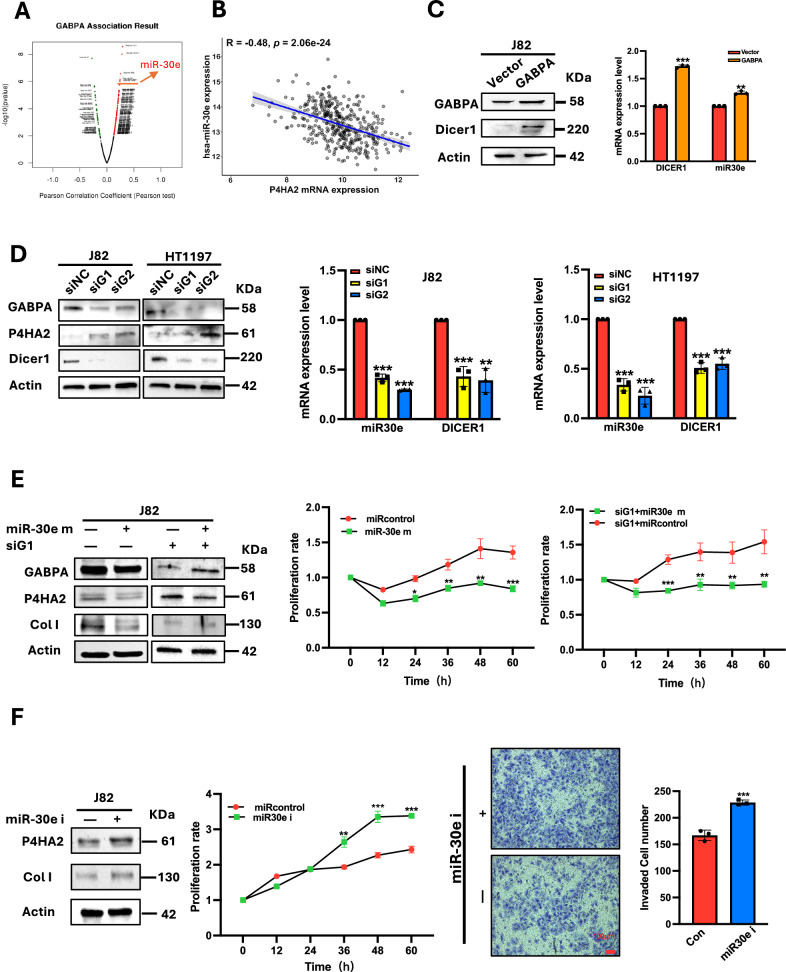
GABPA-upregulated miR-30e expression targets P4HA2 in BC cells. **A** miR-30e is among the top-expressed miRNAs in GABPA-high BC tumors. The TCGA BLCA tumors were divided into GABPA-high and low groups using a median value as the cutoff, and differentially expressed miRNAs were then identified between the two groups. **B** miR-30e levels are anti-correlated with P4HA2 expression in the TCGA BLCA tumors. **C** GABPA overexpression upregulates Dicer1 and miR-30e expression in J82 cells. Left: Immunoblotting and right: qRT-PCR analyses. **D** GABPA depletion downregulates Dicer1 and miR-30e expression in J82 and HT1197 cells. Left: Immunoblotting, middle and right: qRT-PCR analyses. **E** miR-30e mimics (miR-30e m) inhibit P4HA2 expression, Col I formation, and proliferation in J82 cells. Control and GABPA-depleted J82 cells were transfected with miR-30e m and then examined for P4HA2 and Col I level (left panel), and proliferation (middle and right panel). **F** miR-30e inhibitor (miR-30e i) treatment induces P4HA2 expression, Col I formation (Left), enhanced proliferation (middle), and invasion (right) of J82 cells. *, **, and *** indicate *P* < 0.05, 0.01, and 0.001, respectively.

GABPA in general activates target gene transcription [20], and thus, the P4HA2 inhibition mediated by GABPA is unlikely due to a direct transcriptional effect. We have recently identified that DICER1, an endoribonuclease responsible for miRNA maturation, is the GABPA target gene in thyroid cancer (TC) cells, and many miRNAs were positively correlated with GABPA expression in primary TC tumors [28]. These findings indicate the possibility of the GABPA-mediated P4HA2 inhibition via miRNAs. To probe this issue, we searched for miRNAs regulated by GABPA in the TCGA BLCA tumors. >300 miRNAs were identified to correlate positively with GABPA (Fig. 7A), and among the top-ranking miRNAs (miR-151, miR-3913-1, miR-940, miR-98, miR-135a-1, miR-106b, miR-30e, and 30c-1), we paid special attention to miR-30e, as it was previously shown to inhibit P4HA2 expression in hepatocellular carcinoma cells [29]. Consistently, a strongly inverse correlation between miR-30e and P4HA2 expression was observed in BLCA tumors (TCGA cohort) (Fig. 7B). We further determined miR-30e expression in GABPA-overexpressed J82 cells and observed significant miR-30e upregulation in these cells (Fig. 7C). In contrast, downregulated miR-30e expression occurred in GABPA-depleted J82 and HT1197 cells (Fig. 7D). As expected, the altered miR-30e levels were accompanied by the corresponding changes in DICER1 expression in these cells (Fig. 7B–D). Moreover, the direct treatment of J82 cells with miR-30e mimics (miR-30e m) led to reduced P4HA2 expression in control cells (Fig. 7E left) and abolished the increased P4HA2 level resulting from GABPA knockdown (Fig. 7E right). The same scenarios took place in Col I expression (Fig. 7E). The effect of miR-30e m on J82 cell proliferation was evaluated simultaneously, and in both control and GABPA-depleted cells, miR-30e m significantly inhibited cell proliferation (Fig. 7E right). In contrast, when miR-30e inhibitors (miR-30e i) were transfected into J82 cells, P4HA2 and Col I expression were upregulated, accompanied by accelerated cell proliferation and enhanced invasion (Fig. 7F). Thus, miR-30e expression regulated by GABPA is responsible for P4HA2 repression in BC cells.

### P4HA2 and miR-30e expression are associated with advanced BC and patient survival

**Fig. 8:**
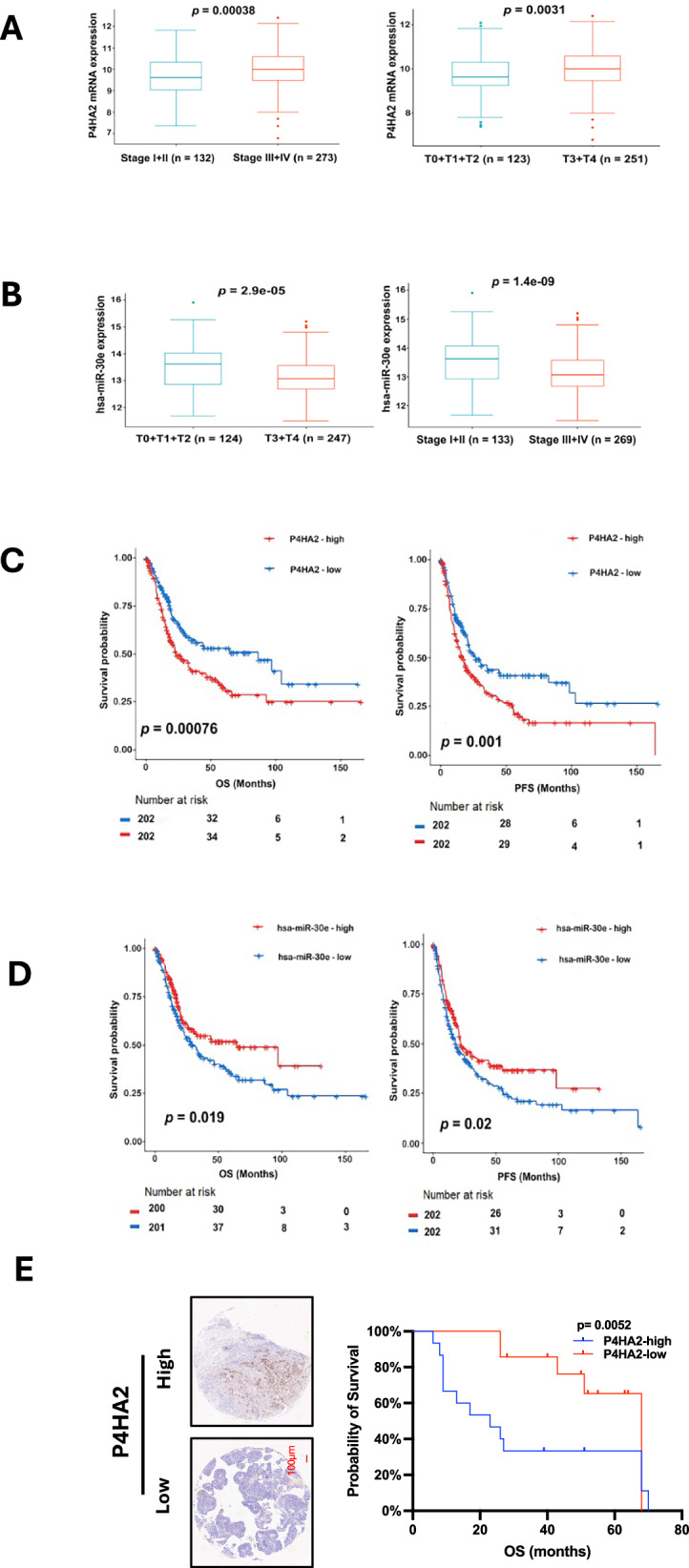
P4HA2 and miR-30e expression are associated with BC progression and patient outcomes. **A**–**D** TCGA BLCA cohort analyses. **A** BC tumor at advanced stages and higher grades expresses significantly increased P4HA2 mRNA. **B** miR-30e expression was significantly downregulated in BC tumors at advanced stages and high grades. **C** K-M plots showed that higher P4HA2 mRNA expression was associated with significantly shorter overall and progression-free survival (OS and PFS) in BLCA patients. **D** Higher miR-30e expression is associated with longer OS and PFS. **E** Higher protein levels of P4HA2 predict significantly shorter OS in our cohort of BC patients. Left: representative IHC staining of P4HA2, and right: K-M plot showing significant association between higher P4HA2 expression and shorter OS.

Finally, we evaluated the clinical association of P4HA2 and miR-30e in BCs. The TCGA BLCA cohort was first analyzed using RNA sequencing data. P4HA2 expression was significantly higher while miR-30e levels were lower in advanced stages and grades of BLCA tumors (Fig. 8A, B). For the effect of P4HA2 on overall and progression-free survival (OS and PFS), patients were divided into P4HA2-high and low two groups using the median expression value as a cutoff. The patients in the P4HA2-high group had significantly shorter OS and PFS compared to the low group patients (Fig. 8C). As expected, miR-30e expression exerted an opposing effect on OS and PFS: Higher levels of miR-30e were associated with significantly longer OS and PFS in BC patients (Fig. 8D).

We further analyzed the association between patient survival and P4HA2 protein expression. In our BC cohort of 45 patients, OS information was available, and their tumors were assessed for P4HA2 expression using IHC. As shown in Fig. 8E, higher P4HA2 levels predicted shorter OS, consistent with the results from RNA data analysis of the TCGA BLCA patients.

## Discussion

The tumor ECM includes the non-cellular components of TME that provide physical scaffolding for the interaction of tumors with stroma and signaling [2]. In BCs, the aberrantly increased ECM remodeling leads to disease invasiveness [15–17]. For instance, lymphatic metastases, associated with extremely poor outcomes in BCs, have been shown to be driven by the aberrant TME, ECM, and rewired transcriptional or other signalings [30–34]. Therefore, elucidating the mechanism(s) underlying ECM dysregulation will provide profound insights into the BC pathogenesis and contribute to the rational development of novel ECM-based strategies for BC therapy. In the study presented herein, we demonstrate that GABPA plays a critical role in modulating ECM depositions. Mechanistically, GABPA inhibits P4HA2 expression, thereby reducing collagen cross-linking, whereas GABPA downregulation promotes ECM remodeling, increases tumor stiffness, and results in the acquisition of invasive phenotypes via the mechanotransduction pathway.

ECM remodeling, attributable to both tumorous and stromal cells, plays multi-faceted roles in carcinogenesis through biochemical and biomechanical mechanisms. Biochemical ECM dysregulation is known to promote cancer development and progression, while biomechanical alterations within tumor tissue are equally or even more important contributors to cancer hallmarks [1–3]. ECM serves as a platform for cross-talk between tumor cells and stroma, and provides a physical scaffold to maintain tissue architecture and tissue-specific function on the one hand, and activates oncogenic pathways via the mechanotransduction signaling on the other [1–3]. The Hippo pathway transcription factors YAP1/TAZ are well-defined downstream effectors in the ECM-mediated mechanotransduction signaling. Activation of the YAP1/TAZ pathway promotes cell proliferation, cancer stem cell functions, and EMT [17, 25, 26, 35, 36]. Consistently, either GABPA depletion or P4HA2 overexpression led to increased collagen formation in BC cells, and nuclear translocation of YAP1 occurred, which was further accompanied by enhanced proliferation, invasion, and EMT marker induction. Importantly, we observed similar in vivo alterations in the murine model, and robustly increased metastasis occurred in the mice that received BC-derived J82 cells with P4HA2 overexpression. In contrast, ectopic GABPA expression inhibited in vivo ECM remodeling and dissemination. These different metastatic abilities are closely associated with ECM stiffness in metastasized tumors.

We have previously demonstrated that the urothelium differentiation transcription factors FoxA1 and GATA3 are the direct target genes of GABPA in BCs [10]. By inducing FoxA1 and GATA3 expression, GABPA promotes differentiation of BC cells, thereby inhibiting disease aggressiveness. In BC tumors, GABPA expression is positively correlated with the luminal subtype characterized by differentiation, whereas inversely correlated with the basal subtype enriched with stem cell and EMT marker expression [10]. Based on our present findings, the stimulatory effect of GABPA on BC cell differentiation, documented in the previous report, may result partially from its regulation of ECM remodeling. GABPA downregulation leads to P4HA2 upregulation, which promotes collagen cross-linking and ECM deposition. Consequently, the increased ECM stiffness provides a niche to inhibit cellular differentiation while supporting maintenance of the basal BC phenotype.

GABPA, as the ETS transcription factor in general, stimulates the target gene transcription. We thus hypothesize that GABPA-mediated P4HA2 inhibition does not result from its transcriptional regulation directly. In recent studies, we and others identified that DICER1 was the direct target gene of GABPA in thyroid carcinoma cells, and many miRNAs were positively correlated with GABPA expression [28, 37, 38]. By analyzing differentially expressed miRNAs between GABPA-high and low tumors in the TCGA BLCA cohort, we observed that miR-30e was among the top ones that positively correlated with GABPA expression, while miR-30e has been shown to target P4HA2 in several cancer types [29]. Consistently, cellular assessments demonstrated that GABPA-mediated inhibition of P4HA2 resulted from its regulation of DICER1 and miR-30e expression. GABPA overexpression and depletion facilitated and repressed DICER1/miR-30e expression, respectively, which consequently led to the corresponding alterations in P4HA2 expression. Thus, the GABPA-DICER1-miR-30e axis controls P4HA2 expression and subsequent ECM remodeling in BCs. On the other hand, the aberrant repression of the DICER1-miR-30e axis was identified in other tumors and non-malignant diseases where miR-30e targeted different molecules [39, 40]. Therefore, it is possible that miR-30e inhibits several oncogenic factors to impede BC progression.

The role for GABPA in oncogenesis is context or cancer-type-dependent. In BCs and RCCs, GABPA may function as a tumor suppressor. Like other tumor suppressors, the aberrant GABPA promoter methylation occurs frequently in BCs and RCCs, which leads to its downregulation in these tumors [10, 41]. However, GABPA is a key transcription factor to transactivate the mutated promoter of the *TERT* gene for telomerase activation. Most BC tumors harbor TERT promoter mutations through which TERT expression is induced [10, 21, 42, 43]. It is well established that TERT is a potent driver for cancer development and progression [18]. We did observe that GABPA depletion resulted in diminished TERT expression in BC cells, while despite so, these cells still exhibited significantly enhanced proliferation and invasiveness. Taken together, GABPA inhibition-mediated effects are more robust and override the influence of TERT downregulation in BC tumors.

In addition to catalyzing the formation of 4-hydroxyproline residues, P4HA2 was recently identified to form a positive feedback loop with HIF1α in BCs [44]. HIF1α directly binds to the P4HA2 promoter to activate gene transcription, while P4HA2 in turn stabilizes HIF1α, which consequently leads to drug resistance [44]. Our results reveal that the GABPA-DICER1-miR-30e axis-regulation of P4HA2 expression plays a key role in ECM remodeling for BC progression, but it remains possible that P4HA2 and/or miR-30e contribute to BC pathogenesis through other mechanisms. Nevertheless, our findings demonstrate that both P4HA2 and miR-30e predict OS and PFS in BC patients. Moreover, the P4HA2 inhibitor 1, 4-DPCA was shown to abolish the invasive phenotype of BC cells, providing a rationale to target P4HA2 for BC treatment. Recently, Wang et al. further observed that aspirin inhibited P4HA2 in hepatocellular carcinoma cells [45]. It will be worth testing the therapeutic effect of aspirin combined with established treatment on BC recurrence/metastasis. Thus, these results are clinically important.

During the preparation of our manuscript, Yang et al. reported that ETV4, another ETS transcription factor, served as a mechanical transducer in human embryonic stem cells [46]. Mechanical stress results in the repression of ETV4 expression, promoting stem cell differentiation via the mechanotransduction signal [46]. It is well established that the ETS family members play essential roles in development, cell survival, and differentiation [20]. The novel function of GABPA and ETV4 in the mechanotransduction signaling, presented here and by Yang et al., provides new mechanistic insights into physiological and pathological processes mediated by these ETS factors. Interestingly, Zhang et al. recently showed that ETV4-mediated tumor-associated neutrophil infiltration stimulated lymphangiogenesis and lymphatic metastasis in BCs [32]. Likely, ETV4 plays important roles in BC progression by regulating ECM and TME.

In summary, we uncover a novel function of GABPA in regulating ECM deposition and mechanotransduction signaling through which BC progression is inhibited. GABPA represses P4HA2 expression and collagen formation through the DICER1-miR-30e circuit, thereby inhibiting ECM deposition and BC aggressiveness. GABPA downregulation occurs frequently in primary BC tumors, which is expected to promote ECM deposition and consequently disease progression. These findings thus unravel a novel role for GABPA in the BC pathogenesis. Moreover, P4HA2 and miR-30e serve as useful prognostic factors for BC. Finally, the direct suppression of P4HA2 activity results in reduced BC cell invasion, and given the inhibitory effect of aspirin on P4HA2 [45], it may be worth evaluating the efficacy of the combination of aspirin with commonly used drugs for BC tumors with low expression of GABPA or miR-30e. Taken together, the present findings have biological and therapeutic implications.

## Supplementary information

Supplemental materials and reagents

Supplemental figures

Supplemental figure legends

## Data Availability

The TCGA data were from (http://cancergenome.nih.gov/).
