# Supplementary material for: The ETS transcription factor GABPA inhibits bladder cancer aggressiveness by repressing extracellular matrix deposition and mechanotransduction signaling
Source: Cell Death Dis. 2025 Aug 14;16(1):618. doi: 10.1038/s41419-025-07935-z (PMC12354829; doi:10.1038/s41419-025-07935-z)
Supplement: Supplementary file 1 — Supplemental materials and reagents [file 41419_2025_7935_MOESM1_ESM.pdf]

Methods

Cell lines and cell culture

BC cell lines used in the present study included J82, HT1197 and SW1710. Cells were cultured in RPMI-1640 medium (Thermo Fisher Scientific, Waltham, MA) supplemented with 10% fetal bovine serum (FBS) (Thermo Fisher Scientific), 100 U/ml penicillin, 100 μg/ml streptomycin and 4 mM L-glutamine. All three cell lines harbor the TERT promoter mutation (C228T), as assessed in the previous study. All cell lines were tested to ascertain mycoplasma-free.

SiRNA and miR-30e inhibitor/mimics transfection

siRNAs specifically targeting GABPA were from Thermo Fisher Scientific. P4HA2 siRNAs, miR-30e inhibitors and mimics were from Integrated DNA Technology (San Diego, CA). They were transfected into cells with Lipofectamine3000 (Thermo Fisher Scientific) according to the protocol provided by the manufacturer. The oligo sequences are listed in Supplementary Table S1.

GABPA and P4HA2 expression vectors and infection

Lentiviral vectors expressing GABPA and P4HA2 were purchased from Abiotech (Jinan, China) and BC cell infection was carried out. After puromycin selection for a week, stably GABPA- or P4HA2-overexpressing cells were obtained.

Cell proliferation assay

Proliferation of BC-derived cells with different treatments was monitored and analyzed real-time for up to 72 hours using IncuCyte S3 Live-Cell Analysis System (Essen Bioscience, Ann Arbor, MI). Changes in the phase area confluence represent cell proliferation.

RNA extraction, reverse transcription and qRT-PCR

Total RNA was extracted with Trizol-Reagent (Thermo Fisher Scientific), and reversely transcribed using a High-Capacity cDNA Reverse Transcription Kit (Thermo Fisher Scientific). qPCR was performed in QuantStudio 7 Flex Real-Time PCR System using SYBR Green (Thermo Fisher Scientific). Levels of target mRNA (GABPA, P4HA2, TERT, VIM) were calculated based on the ΔCT values and normalized to human β2-M expression. For miR-30e assessment, RNA was reversely transcribed using MicroRNA first-strand synthesis and

miRNA quantitation kit (#638315, Takara, Kyoto, Japan). Primers used in this study are documented in Supplementary Table S1.

Immunoblotting

Cellular proteins were extracted using Pierce RIPA Buffer (Thermo Fisher) with 1% Phenylmethanesulfonyl fluoride (Sigma-Aldrich) and quantified with DC Protein Assay (Bio-Rad). Thirty μg of proteins were separated in Mini-PROTEAN TGX Gels (Bio-Rad) and transferred to PVDF membranes using Trans-Blot Turbo Transfer Pack (Bio-Rad). Membranes were blocked with 5% non-fat milk diluted in TBST, and then incubated with primary antibodies and secondary antibodies before imaged with Clarity Max Western ECL Substrate (Bio-Rad, 1705062) and ChemiDoc MP Imaging System (Bio-Rad). Primary antibodies used for cell lines included anti-GABPA (Santa Cruz, sc-22810), anti-P4HA2, (Proteintech, 13759-1-AP), anti-Col I (NOVUS, NB6000-408), anti-Col III (Proteintech, 22734-1-AP), VIM (Proteintech, 10366-1-AP), anti-YAP1 (Santa Cruz, sc-101199), anti-DICER1 (Cell signaling technology, D38E7), anti-GAPDH ((Proteintech, 60004-1) and anti-β-Actin (Santa Cruz, sc-47778). Secondary antibodies include Goat Anti-Mouse IgG (H+L)-HRP Conjugate (Bio-Rad, 170-6516) and Goat Anti-Rabbit IgG (H+L)-HRP Conjugate (Bio-Rad, 170-6515).

Cellular invasion assays

Matrigel-coated invasion assays were used. Fifty μl of matrigel (Corning Life Sciences, Flintshire, UK) was loaded to the bottom of the upper chamber, and cells (1.0×10^4) were then seeded into the upper chamber. The low chamber contained RPMI-1640 medium with 20% FBS. Cells passing through the matrigel were stained with crystal violet, counted, and photographed 24 hours later.

Collagen gradient stiffness cell culture

Genipin-crosslinked collagen gels with defined stiffness were prepared under sterile conditions, as described by Ishihara et al (Reference 27). Briefly, Col I (CLS354249, Merck) was dissolved (5mg/ml) and then mixed with 2 mM genipin (G4769, Merck) in HEPES buffer (pH 7.4) to final genipin concentrations of 0 mM, 0.1 mM, and 1 mM, corresponding to gel stiffness values 0.0292 ± 0.003 kPa, 1.49 ± 0.12 kPa, and 9.20 ± 0.45 kPa, respectively. The mixtures were cast into 6-well plates (2 mL/well) and incubated at 37°C in a humidified 5% CO_2 incubator for 72 h to complete polymerization. Upon gelation, gels were equilibrated in DMEM supplemented with 10% fetal bovine serum (FBS) and 1% penicillin-streptomycin for 24 h at 37°C to remove

unreacted genipin and stabilize the matrix. Cells were seeded onto the genipin-collagen gels at a density of 2 × 10^4 cells per well in complete DMEM medium. Cell proliferation was monitored by phase-contrast microscopy （Millicell® Digital Cell Imager） at 24h, 48h post-seeding, and confluence was quantified with threshold-based segmentation. Cells were dissociated from the gels via enzymatic digestion with collagenase type I (17018029, Thermo Fisher; 3 mg/mL) and 0.05% trypsin-EDTA for 15 min at 37°C. The cell suspensions were collected for other analyses.

Immunofluorescence

Control and GABPA-depleted J82 cells were treated with 4% paraformaldehyde followed by 5% BSA. The cells were then incubated with a primary anti-YAP1 ab (Santa Cruz) at room temperature for 2 hours followed by further incubation with a secondary chicken anti-rabbit ab conjugated with Alexa Fluor™480 (#21411, Thermo Fisher Scientific). Cells were finally counterstained with DAPI (D1306, Thermo Fisher) and imaged under a fluorescence microscopy. The fluorescence intensity ratio between nucleus and cytoplasm was evaluated.

For primary BC tumors, a multiplex fluorescent immunohistochemistry kit (Panovue Biosciences, 10001100050) was applied with Tyramide signal amplification (TSA) method. Slides were deparaffinized and rehydrated followed by antigen-retrieval using citric acid buffer. Endogenous peroxidase was deactivated by H_2O_2. Goat serum (10%) was used to block non-specific binding. Slides were then sequentially incubated overnight at 4 °C with the following primary antibodies: GABPA (21542-1-AP, proteintech), P4HA2 (13759-1-AP, proteintech), and Col I (14695-1-AP, proteintech). GABPA was visualized by Alexa Fluor 488-conjugated anti-rabbit IgG staining, while P4AH2 and Col I were by Alexa Fluor 594-conjugated anti-rabbit IgG. DAPI (1 μg/mL, blue fluorescence) was used for nuclear counterstaining. Finally, slides were mounted with Prolong Gold Antifade Mountant and imaged using a confocal microscope (AxioObserver Z1). Fluorescence intensity for GABPA (green), P4HA2 (red) and Col I (red) was quantified using image J.

The TCGA BC cohort and reactome gene set analyses

The RNA sequencing and other-related data for BC cases within the TCGA database were downloaded at the TCGA Research Network (http://cancergenome.nih.gov/) in Oct. 2022. The RNA-seq data processing was performed using the R software packages limma and maftools.

Spearman analysis was used to assess the expression correlation. Reactome analyses were performed using gene sets from the Reactome database.

GABPA-transgenic (GABPA-T) mice, nude mice and tumor cell injection

GABPA-T mice were made from C57B strain (Shanghai Nanfang Moshi Biology Research Center, Shanghai, China). Six-week-old male athymic BALB/c nude mice were purchased from Beijing Vital River Laboratory Animal Technology Co., Ltd./Charles River Laboratories, Beijing, China, and used to evaluate the *in vivo* effect of GABPA and P4HA2 on metastasis. J82 cells expressing ectopic GABPA (J82/GABPA), P4HA2 (J82/P4HA2) or both (J82/GABPA/P4HA2) and control cells with empty vectors (J82/Control) were injected into nude mice via the tail vein. Five mice were included in each group. Mice were killed after 10 weeks and lungs/livers were collected for evaluation of tumor seeding or metastasis by using BOUNIS/hematoxylin and eosin (H&E) staining. The study was approved by Shandong University Second Hospital Ethics Committee and Shandong Provincial Hospital Ethics Committee (#KYLL-2017(KJ)A-0001 and 2020-009).

PSR staining of mouse dermis

Paraffin-fixed skin tissue (from mouse back) sections were dewaxed and hydrated. The sections were first stained with haematoxylin followed by incubation with PSR solution. Under a light microscope, collagen fibers are red against a yellow background. Polarization analysis was carried out using a NIKON Eclipse ci upright microscope (NIKON digital sight DS-FI2) and software NIS_F_Ver43000_64bit_E. Under polarized light microscopy, collagen I (Col I) is thick fibers orange-yellow or bright red, whereas collagen III (Col III) is thin green fibers.

AFM measurement

Tumor ECM stiffness from the xenograft mouse model was quantified using AFM. paraffin-embedded tumors were deparaffinized, rehydrated, and dried. The measurement was carried out by a commercial AFM setup (NT-AIST, HORIBA, Japan) in the force mapping mode using a high-quality tip (MikroMasch, USA). The spring constant of the cantilever was 5 N/m. The tips were cleaned with ethanol and UV light to remove the contaminations after each force map. The topography and Young’s modulus image were recorded to identify the bio-samples structures. To evaluate the Young’s modulus from the samples, the Young’s modulus maps on a scan area of 10 μm × 10 μm with 20 × 20 force-indentation curves were determined.

Primary and xenografted BC tumors and IHC

Primary BC tumors in the tissue microarray (TMA) were obtained from Shanghai Outdo Biotech (Shanghai, China) and a total of 45 specimens were included. Patient clinical characteristics with OS data (dead or alive after 8 years follow-up) are listed in Table S2. The study was approved by Shandong University Second Hospital Ethics Committee (#KYLL-2017(KJ)P-0002). Informed consent was obtained from all the patients. Tumors were analyzed for GABPA and P4HA2 expression using IHC. Tumor tissues in the TMA were deparaffinized and rehydrated followed by antigen-retrieval using citric acid buffer. Endogenous peroxidase was deactivated by H_2O_2. Goat serum (10%) was used to block non-specific binding and the corresponding primary antibodies (GABPA and P4HA2, from Proteintech, 21542-1-AP and 13759-1-AP, respectively) were then added for overnight incubation at 4°C. After incubation with secondary antibodies for 45 mins at room temperature, DAB staining (Thermo Fisher Scientific) was used to detect the antigen-antibody binding. The slides were examined by two of the co-authors (MD and DX) and mean values of GABPA and P4HA2 positive cells were presented based on the results from two observers. For each tumor, a total of 200 cells in two fields were analyzed.

Metastatic tumors in mouse lungs were analyzed for GABPA, P4HA2, and Ki-67 using IHC. Antibodies targeting the molecules above were all from Proteintech.

Statistical analyses

All statistical analyses were performed using IBM SPSS Statistics version 24 (IBM, Armonk, NY). Based on the distribution of data, Student's t-test, Mann-Whitney U-test, and Chi^2-test or Fisher's exact test were used. Spearman's Rank-Order Correlation was applied to determine correlation coefficient ρ and P value. OS and PFS were visualized with Kaplan-Meier plots. Survival analyses were performed with log-rank test. P-values < 0.05 were considered statistically significant.

Table S1. Sequences of primers, siRNAs and plasmids used in the study.

TERT	Forward	5’-CGG AAG AGT GTC TGG AGC AA-3’
	Reverse	5’-GGA TGA AGC GGA GTC TGG A-3’
GABPA	Forward	5’- AAGAACGCCTTGGGATACCCT-3’
	Reverse	5’- GTGAGGTCTATATCGGTCATGCT-3’
YAP	Forward	5’-TAGCCCTGCGTAGCCAGTTA -3’
	Reverse	5’-TCAT GCTTAGTCCACTGTCTGT -3’
P4HA2	Forward	5’-GCCTGCGCTGGAGGACCTTG -3’
	Reverse	5’-TGT GCCTGGGTCCAGCCTGT -3’
VIM	Forward	5-' CTC TTC CAA ACT TTT CCT CCC-3'
	Reverse	5-' AGT TTC GTT GAT AAC CTG TCC-3'
DICER1	Forward	5-'CCT AGA CCA CCC CTA TCG AGA -3'
	Reverse	5-' CAG GTC AGT TGC AGT TTC AGC A-3'
miR-30e 5P		5’-ACACTCCAGCTGGGTGTAAACATCCTTG-3’
β2-M	Forward	5’-GAA TTG CTA TGT GTC TGG GT-3’
	Reverse	5’-CAT CTT CAA ACC TCC ATG ATG-3’
P4HA2（mice）	Forward	5’-TCC AGT TTG TAC GTG TCC TGA-3’
	Reverse	5’-GAC CTT GTC CTT CAG GAT GCT-3’
siRNA GABPA (siG1)		5'-GGA GCU GAU AGA AAU UGA GAU UGA U-3' (sense)
		5’- AUC AAU CUC AAU UUC UAU CAG CUC C-3’ (anti-sense)
siRNA GABPA (siG2)		5'-GCA GAG UGC ACA GAA GAA AGC AUU G-3’ (sense)
		5’-CAA UGC UUU CUU CUG UGC ACU CUG C-3’ (anti-sense)
hs.Ri. P4HA2.13.1	Forward	5'-AAAGUUCUGAUACCUUGUUUACATG-3'
	Reverse	5'-CAUGUAAACAAGGUAUCAGAACUUUGG-3'
hs.Ri. P4HA2.13.2	Forward	5'-CAACAGAAGUUGACUGACAUCCUTT-3'
	Reverse	5'-AAAGGAUGUCAGUCAACUUCUGUUGAU-3'
miR-30e mimics 5P	sense	5-' UGUAAACAUCCUUGACUGGAAG-3'
	antisense	5-'CUUCCAGUCAAGGAUGUUUACATT-3'
miR-30e inhibitor 5P		5-' AACUUCCAGUCAAGGAUGUUUACA -3'
Si-NC	sense	5-'UUCUCCGAACGUGUCACGUTT -3'
	antisense	5-'ACGUGACACGUUCGGAGAATT-3'

Constructs

Lenti-CMV-MCS-EF1a-mOrange-T2A-BSD-P4HA2	Abiotech, jinan, china
Lenti-CMV-MCS-EF1a-GFP-T2A-Puro-GABPA	Abiotech, jinan, china

Table S2. Clinicopathologic characteristics of 45 BC* patients

Variable		Number
**Sex**		
	Male	37
	Female	8
**Age at diagnosis (mean ± SD)**		70.0 ± 9.0
**TNM stage**		
	<T2	8
	≥T2	34
**Grade**		
	Low	2
	High	43
**Invasion**		
	NMIBC**	3
	MIBC***	40
**Tumor size (cm)**		
	< 3	8
	≥ 3	35
**Tumor number**		
	Single	39
	Multiple	4

*BC, Bladder cancer.

**NMIBC, Non-muscle invasive bladder cancer.

***MIBC, Muscle invasive bladder cancer.
