# Supplementary figures and images for: The ETS transcription factor GABPA inhibits bladder cancer aggressiveness by repressing extracellular matrix deposition and mechanotransduction signaling
Source: Cell Death Dis. 2025 Aug 14;16(1):618. doi: 10.1038/s41419-025-07935-z (PMC12354829; doi:10.1038/s41419-025-07935-z)

# Supplemental Figure S1.

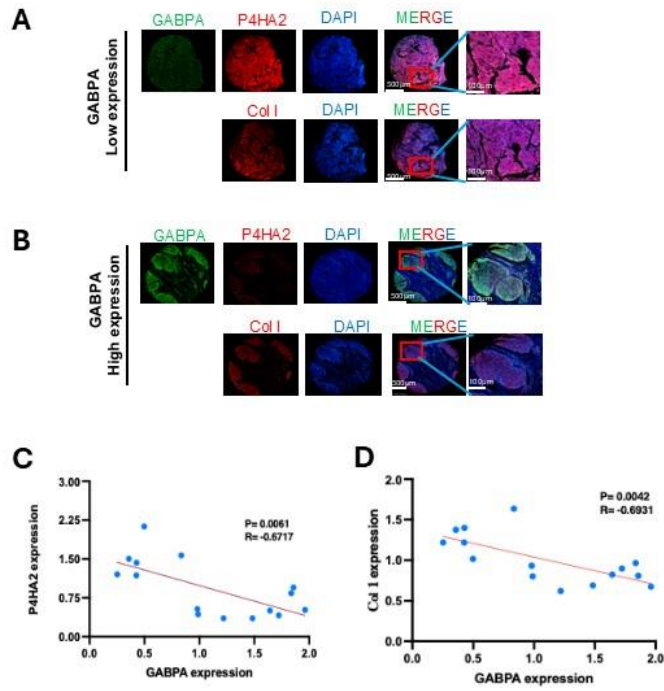

Supplemental Figure S2.

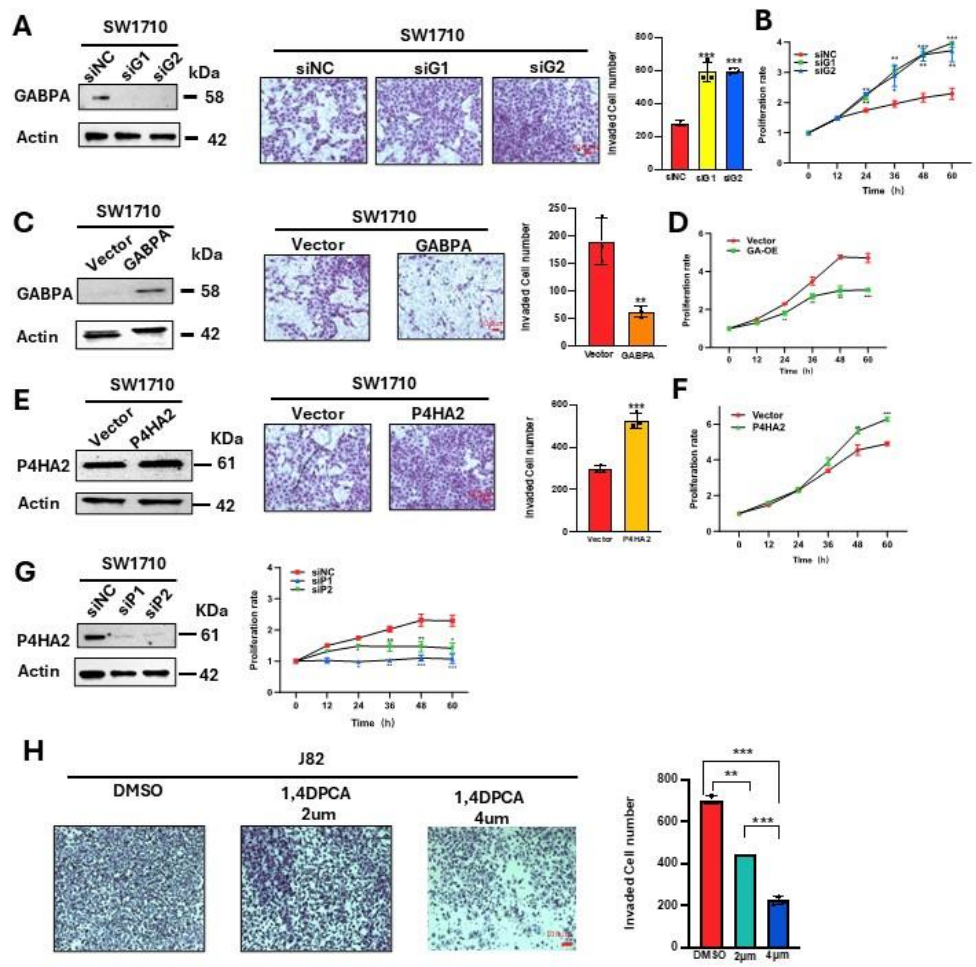

Supplemental Figure S3.

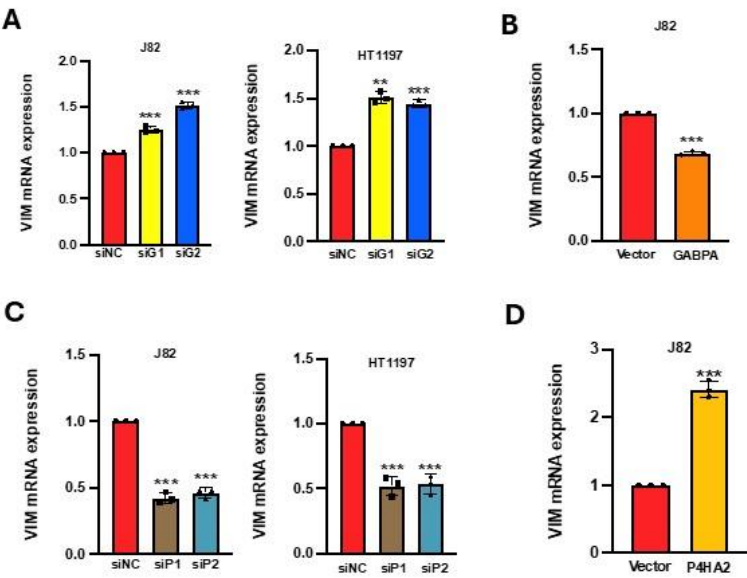

Supplement: Supplementary file 2 — Supplemental figures [file 41419_2025_7935_MOESM2_ESM.pdf]
