# Supplementary material for: The ETS transcription factor GABPA inhibits bladder cancer aggressiveness by repressing extracellular matrix deposition and mechanotransduction signaling
Source: Cell Death Dis. 2025 Aug 14;16(1):618. doi: 10.1038/s41419-025-07935-z (PMC12354829; doi:10.1038/s41419-025-07935-z)
Supplement: Supplementary file 3 — Supplemental figure legends [file 41419_2025_7935_MOESM3_ESM.pdf]

Supplemental Figure S1. Inverse correlation of GABPA with P4HA2 and Col I in primary BC tumors. Expression levels of GABPA (green), P4HA2 (red) and Col I (red) were quantified using multi-color immunofluorescence (FI) with Tyramide signal amplification in 15 primary BC tumors. (A) and (B) Representative images of GABPA-low and high tumors, respectively. Inset areas were enlarged in the right panels. (C) and (D) Inverse correlation of GABPA with P4HA2 and Col I levels, as quantified based on fluorescence densities of each molecule.

Supplemental Figure S2. Opposing effects of GABPA and P4HA2 on invasion and proliferation of BC cells. (A) SW1710 cells were transfected with two different GABPA siRNAs (siG1 and siG2) and then analyzed for their invasion ability. (B) SW1710 cells in (A) were analyzed for their proliferation. (C) and (D) SW1710 cells with ectopic GABBPA expression were analyzed for their invasion and proliferation abilities, respectively. (E) and (F) SW1710 cells with ectopic P4HA2 expression were analyzed for their invasion and proliferation abilities, respectively. (G) SW1710 cells were transfected with two different P4HA2 siRNAs (siP1 and siP2) and then analyzed for their proliferation. (H) J82 cells were treated with P4HA2 inhibitor 1,4DPCA at two different concentrations and cellular invasion ability were then analyzed. ** and *** indicate $P < 0.01$ and 0.001, respectively.

Supplemental Figure S3. Opposing effects of GABPA and P4HA2 on Vimentin (VIM) mRNA expression in BC cells. The level of VIM mRNA was assessed using qRT-PCR. (A) J82 and HT1197 cells were transfected with GABPA siRNAs (siG1 and siG2) and cells were then analyzed for VIM expression. (B) J82 cells with ectopic GABPA expression were analyzed for VIM mRNA levels. (C) J82 and HT1197 cells were transfected with P4HA2 siRNA (siP1 and siP2) and cells were then analyzed for VIM expression. (D) J82 cells with ectopic P4HA2 expression were analyzed for VIM mRNA levels. ** and *** indicate P < 0.01 and 0.001, respectively.
